# Chatbot for Social Need Screening and Resource Sharing With Vulnerable Families: Iterative Design and Evaluation Study

**DOI:** 10.2196/57114

**Published:** 2024-07-19

**Authors:** Emre Sezgin, A Baki Kocaballi, Millie Dolce, Micah Skeens, Lisa Militello, Yungui Huang, Jack Stevens, Alex R Kemper

**Affiliations:** 1 Nationwide Children's Hospital Columbus, OH United States; 2 Centre for Health Informatics, Australian Institute of Health Innovation, Macquarie University Sydney Australia; 3 The Ohio State University Columbus, OH United States

**Keywords:** social determinants of health, social needs, chatbot, conversational agent, primary care, digital health, iterative design, implementation, evaluation, usability, feasibility

## Abstract

**Background:**

Health outcomes are significantly influenced by unmet social needs. Although screening for social needs has become common in health care settings, there is often poor linkage to resources after needs are identified. The structural barriers (eg, staffing, time, and space) to helping address social needs could be overcome by a technology-based solution.

**Objective:**

This study aims to present the design and evaluation of a chatbot, DAPHNE (Dialog-Based Assistant Platform for Healthcare and Needs Ecosystem), which screens for social needs and links patients and families to resources.

**Methods:**

This research used a three-stage study approach: (1) an end-user survey to understand unmet needs and perception toward chatbots, (2) iterative design with interdisciplinary stakeholder groups, and (3) a feasibility and usability assessment. In study 1, a web-based survey was conducted with low-income US resident households (n=201). Following that, in study 2, web-based sessions were held with an interdisciplinary group of stakeholders (n=10) using thematic and content analysis to inform the chatbot’s design and development. Finally, in study 3, the assessment on feasibility and usability was completed via a mix of a web-based survey and focus group interviews following scenario-based usability testing with community health workers (family advocates; n=4) and social workers (n=9). We reported descriptive statistics and chi-square test results for the household survey. Content analysis and thematic analysis were used to analyze qualitative data. Usability score was descriptively reported.

**Results:**

Among the survey participants, employed and younger individuals reported a higher likelihood of using a chatbot to address social needs, in contrast to the oldest age group. Regarding designing the chatbot, the stakeholders emphasized the importance of provider-technology collaboration, inclusive conversational design, and user education. The participants found that the chatbot’s capabilities met expectations and that the chatbot was easy to use (System Usability Scale score=72/100). However, there were common concerns about the accuracy of suggested resources, electronic health record integration, and trust with a chatbot.

**Conclusions:**

Chatbots can provide personalized feedback for families to identify and meet social needs. Our study highlights the importance of user-centered iterative design and development of chatbots for social needs. Future research should examine the efficacy, cost-effectiveness, and scalability of chatbot interventions to address social needs.

## Introduction

### Background

Unmet social needs (eg, food insecurity, housing insecurity, transportation challenges, and economic instability) are strongly associated with poor health outcomes [1], perpetuating health inequities [2,3] and informing social determinants of health. Children are especially at risk when families face unmet social needs [4,5]. Driven by recent recommendations, there has been a rapid uptake of social need screening [2,6]. Although screening can be relatively straightforward, linkage to resources to address social needs is a major challenge [7,8].

Typically, clinicians provide families who are identified with a social need with a resource sheet. Families are then responsible for follow-up. Most clinics do not have social workers or other staff to help families access services and overcome barriers, such as language or cultural differences, financial constraints, transportation issues, limited internet access, or lack of awareness about available resources. Thus, families are often left to navigate complex social services independently, which can result in significant difficulties in obtaining much-needed assistance and support [9]. This passive provision of information is rarely effective. It is imperative to develop scalable strategies that screen for social needs and effectively link to services.

Digital health technology could improve both screening and resource referral to assist vulnerable populations [10]. Currently, electronic health records (EHRs) help facilitate screening, and patient portals help with bidirectional communication [2]. However, this does not eliminate the need to maintain lists of resources and the need to link individuals to matching resources. Semiautonomous intelligent and conversational digital health technologies, such as chatbots (conversational agents or dialogue systems), can help address these gaps. By using machine learning algorithms and natural language processing, chatbots can deliver personalized feedback and health recommendations to a wide range of users via interactive, user-friendly interfaces that are designed to maintain human conversation [11,12]. The capacity of the technology to reach and assist a large number of users simultaneously offers a cost-effective and efficient method for delivering personalized health services [13,14]. Chatbots have been used for health care communications, including health information seeking, health screening, and health care support, and to improve adherence to recommended care [15-20]. A previous study [21] described a chatbot to screen adults with low and high health literacy for social needs in emergency departments. The authors reported that the performance of the chatbot is comparable to that of traditional screening, and there is a greater interest from lower literacy participants for a chatbot. At a broader scale, chatbots show promise to facilitate social need screening and provide personalized resources to families outside of the traditional clinic setting via speech or text and could improve access [22,23] and further contribute to increased understandability and personalization while addressing social needs [21,24].

The DAPHNE (Dialog-Based Assistant Platform for Healthcare and Needs Ecosystem) chatbot project has been initiated to address unmet social needs via a conversational interface for low-income or resource-limited families, who often have trouble with a complex web of social challenges that include food insecurity, inadequate housing, and financial difficulties [25]. These vulnerable groups typically experience lower incomes, higher rates of unemployment, and diminished access to quality health care services. In the Nationwide Children’s Hospital (NCH) primary care clinics, approximately 10% of families are identified to have at least 1 unmet social need [4], with >16% facing food insecurity (based on current data from our ambulatory patient population). This emerging need for social support has been the main motivation of our study. In this paper, we report our findings from the iterative design, prototype development, and evaluation of the DAPHNE chatbot for social need screening and resource referral. In this stage of development, we focus on food insecurity, the most frequently endorsed unmet social need, which has a significant impact on health care costs [26,27].

### DAPHNE Chatbot

**Figure 1:**
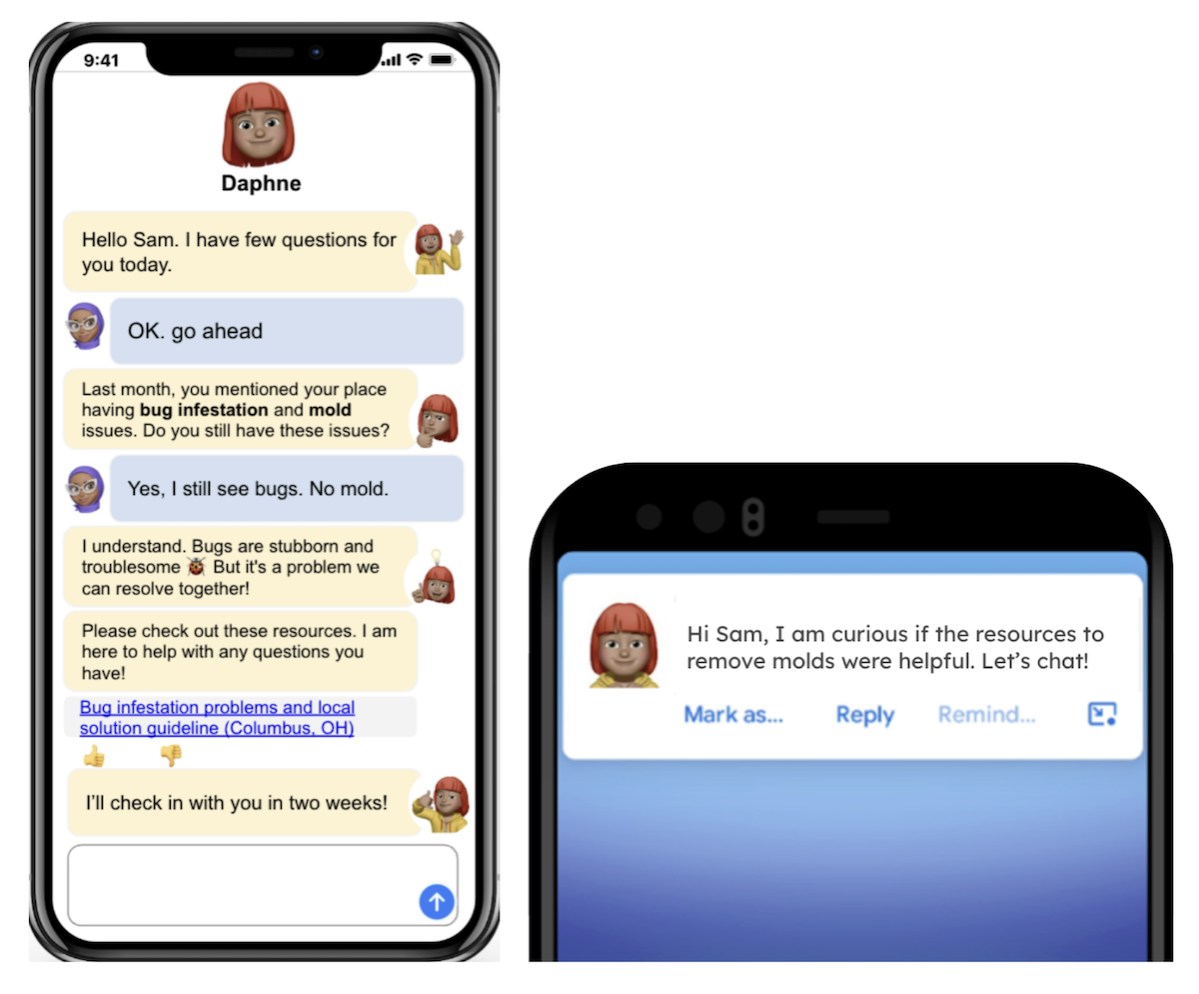
Initial wire frames and mock-ups.

DAPHNE is a web-based application available via a computer or an iOS- or Android-based mobile device over a web browser. Figure 1 presents the initial wire-frame concept. The DAPHNE conversational interface prototype was designed using Adobe XD (Adobe Inc), Expo (650 Industries, Inc), and JavaScript (Oracle Inc) with a secure text-to-speech and speech-to-text service for voice interaction using Amazon Web Services (Amazon.com, Inc). Conversational flow was designed to be rule based. We opted for a rule-based design over pretrained language model or hybrid model at this stage to ensure greater transparency, predictability, and control in system responses, which is crucial for accurately identifying needs and retrieving specified resources.

The architecture, including data storage, conversational intelligence, information search, and referral services, uses Amazon Web Services and Microsoft Azure (Microsoft Corp) backend services. DAPHNE leverages application programming interfaces (APIs) provided by community resource platforms to access resource databases. These platforms, such as FindHelp.org, 211.org, and Cap4Kids.org, provide information about community resources categorized by geographic region. DAPHNE’s architecture is designed to be integrated with EHR, enabling the communication of social need screening results to health care providers such as social workers, community health workers, and care teams. Its functionalities are listed in Textbox 1. In the scope of this study, the resource database of DAPHNE was locally created for testing purposes, without leveraging real-time API connection to the community resource platforms. In addition, the prototype was limited to screen 1 social need to reduce complexity during the testing.

**Figure 2:**
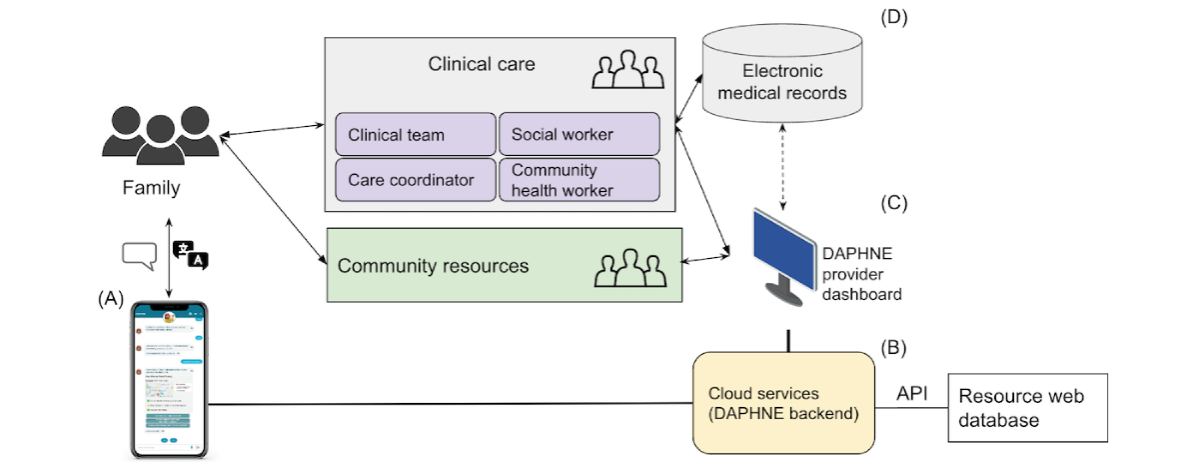
Conceptual framework of the chatbot ecosystem: (A) Conversational interface, (B) Backend cloud services and API connection, (C) provider dashboard, and (D) integration to medical records. API: application programming interface; DAPHNE: Dialog-Based Assistant Platform for Healthcare and Needs Ecosystem.

Figure 2 outlines the chatbot ecosystem framework. Within the scope of this study, we are focusing on iterative design and the evaluation of engagement using conversational interface (Figure 2A). In the next phases, DAPHNE will have backend cloud services and API connection to enable access to web-based resource databases (Figure 2B) and provider dashboard to track engagement, control content (Figure 2C), and integration to medical records to report back social determinants of health monitoring (Figure 2D).

**DAPHNE (Dialog-Based Assistant Platform for Healthcare and Needs Ecosystem) chatbot functionalities and descriptions.**

**Profile page**

- Users create their account and set up profile details, including name, zip code, family type and size, and income level. The information is to be used for resource-finding queries.

**Avatar**

- Users can create an avatar to personalize their chatbot experience. For the prototype, we used Apple’s Memoji to create an avatar that dynamically reflects emotions [28].

**Language selection**

- Users can select their preferred language. The prototype included the following languages: Somali, Nepali, and Spanish.

**Audio narration**

- Users can use the text-to-speech and speech-to-text features to enable audio entry and engagement and listen to the responses.

**Multimodal input**

- Users can use voice input (using speech to input), assistive buttons with prepopulated responses to select, and text entry with a free-text form to interact with the chatbot.

**Social need screening**

- DAPHNE uses the following standardized questions [4] to guide the screening process:
  Food: within the past 6 months, you worried that your food would run out before you had money to buy more.
  Housing: do you think you are at risk of becoming homeless?
  Transportation: in the past 12 months, has lack of transportation kept you from medical appointments or from getting medications?
  Utility: in the past year, has the utility company shut off your service for not paying your bills?

**Interactive resource sharing**

- DAPHNE can search the resource databases and present matching resources based on user response and ask follow-up questions.

**Check-in and reminder notifications**

- DAPHNE can send notifications. Scheduled check-in: it can collect information about whether the resource shared was useful. Reminder: it can set and send reminders asking whether the user would like to engage in another time. (Refer to Figure 1 for a reminder notification example.)

## Methods

### Study Design and Participants

**Figure 3:**
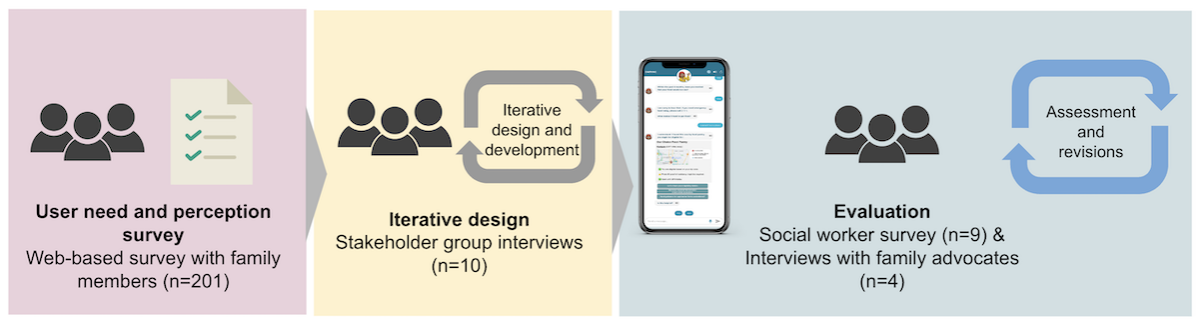
Study process diagram.

The research was reported as a three-stage study: (1) understanding family needs and perception toward chatbots, (2) designing the chatbot, and (3) evaluating its feasibility and usability. We used stepwise, user-centered, iterative, and participatory development and improvement processes to ensure the proposed technology meets needs and expectations. Figure 3 presents the design, development, and evaluation stages.

*Study 1* aims to understand families’ ability to meet social needs and access essential resources and perceptions toward using chatbots to find resources. We conducted a cross-sectional web-based survey. A total of 201 adults in US households participated. Participants were living together with spouses, children, and significant others and self-reported an annual household income of ≤US $29,999 as of August 2023. The participants were recruited through a web-based platform designed for academic and market research, Prolific [29]. We followed a convenience sampling approach, inviting available participants via the survey tool. Survey details are available in Multimedia Appendix 1.

*Study 2* focuses on iterative design, which includes the stages of ideation, prototyping, and refinement [30,31]. We held internet-based sessions with an interdisciplinary group of stakeholders. The sessions focused on answering the following research questions to understand design preferences and needs: “What are the pain points in current practices of social need screening and resource sharing?” “Why should we use or not use technology to facilitate this process?” and “How can we design and use a chatbot to connect with families in primary care settings in order to address social needs effectively?” The interdisciplinary stakeholder group (n=10) was formed internally at the NCH, including an epidemiologist (n=1, 10%), a primary care physician (n=1, 10%), a nurse (n=1, 10%), the director of clinical social work services (n=1, 10%), a community health worker (n=1, 10%), a public health scientist (n=1, 10%), an industry partner leader (n=1, 10%), a community partner leader (n=1, 10%), a family advocate (n=1, 10%), and an information system expert (n=1, 10%). Stakeholder group members were recruited within the NCH network (including primary care clinics) in September 2022.

*Study 3* is a mixed methods evaluation of the prototype. Our evaluation methodology was informed by a feasibility framework [32], technology acceptance model [33], and usability scale—Usability Metric for User Experience–Lite (UMUX-Lite) [34]. We conducted scenario-based usability testing via a focus group interview with community health workers (who are also family advocates as part of the community) and via a web-based survey with social workers to examine the usability and feasibility of DAPHNE (semifunctional prototype) for families and communities. During these sessions, participants interacted with the chatbot to simulate the process of accessing and evaluating social resources (refer to Multimedia Appendix 1 for the scenario and questions). They used the chatbot to enter responses, navigate resource information, and provide feedback on its functionality qualitatively and quantitatively (via UMUX-Lite). Community health workers (n=4) and social workers (n=9) were recruited within the hospital network via email or phone (January to February 2023). Participation was voluntary for all participants.

### Ethical Considerations

This study received ethics board approval (NCH institutional review board #00003766). All participants provided informed consent to participate to the study. Participants did not opt out of the study. Collected data and transcripts were deidentified. Participants received compensation for their participation, if permissible. This paper reports an aggregate summary of the data generated during the study, without any identifiable information. Due to privacy and confidentiality reasons, we are not able to share individual data points or transcripts.

### Data Collection

In study 1, after participants provided consent, they completed a survey about their experiences. The survey captured their experiences and perceptions regarding the accessibility of social need resources. Questions included items on the awareness of and ability to access community support programs, methods used to obtain resources, and openness to using technological tools such as chatbots for resource assistance. Responses were collected anonymously, and the entire data collection process was structured such that the security and confidentiality of the participants were ensured. In study 2, iterative design sessions consisted of interactive interviews with open discussion guided by the research questions and moderated by a researcher. Wire frames were used to communicate initial design and revised designs of the chatbot (Figure 1). In total, three 1-hour sessions of stakeholder interviews were held between September to December 2022. The research team continuously communicated with the stakeholder group via email to share iterative improvements in the prototype. Throughout the sessions and conversations, stakeholder feedback was captured as conversation notes and observational notes. In study 3, social workers completed a 20-minute web-based scenario-based study to use the chatbot prototype and provide feedback (refer to Multimedia Appendix 1 for scenario and survey details). They responded via a web-based survey tool (REDCap [Research Electronic Data Capture]; Vanderbilt University). Community health workers were invited to a single-session focus group interview at the hospital (approximately 1 hour). The study team introduced the chatbot and its functionalities, shared examples, and provided a scenario-based demonstration. Usability questions and questions about dialogue and conversational design, voice interaction, perceived opportunities, and barriers were verbally discussed, which followed a similar approach to the web-based survey protocol. Data were collected via field notes.

### Data Analysis

Study-1 analysis included descriptive statistics to summarize demographic information and responses to survey questions. We compared observed distributions of income, age, and employment status with responses to the questions on the ability to meet social needs, knowledge about community resources, and perception of chatbot use. Then, we conducted chi-square analysis to assess the association and independence of categories. In study 2, we conducted content analysis to inform the chatbot development process [35]. Stakeholder feedback was systematically analyzed by a single researcher to identify emerging themes, patterns, and insights, which were instrumental in understanding stakeholders’ needs and expectations. Given the nature of semistructured interviews and scenario-based surveys, study-3 data were analyzed using thematic analysis to synthesize the qualitative data and to understand the meanings and experiences reported in response to open-ended questions and captured during the interviews [36]. The process began with 2 researchers independently conducting initial coding of the data. This coding was primarily inductive, allowing themes to emerge from the data, although a preliminary framework based on existing literature was also considered to guide the analysis. Regular discussions were held to review codes and themes, ensuring consistency and comprehensiveness. Data saturation was assessed to determine when no new themes were emerging, indicating sufficient depth of inquiry. Discrepancies between researchers were resolved through consensus; if consensus could not be reached, a third researcher was consulted to make a final decision, ensuring objectivity and reliability. In addition, we reported the total score of UMUX-Lite, with an expected usability score of ≥60 [34,37]. The thematic analysis and usability results were triangulated to provide a robust understanding of both user satisfaction and deeper user experiences.

## Results

### Study 1: Family Needs and Perceptions

We surveyed 201 low-income households, each with at least 1 unmet social need, to understand their willingness to use a chatbot for resource assistance. As shown in Table 1, demographic data showed an equal sex split (male: 100/201, 49.8%; female: 100/201, 49.8%; unreported: 1/201, 0.5%). Age distribution skewed toward the 21-40 years range (122/201, 60.7%), and the most reported income level was from US $20,000 to US $29,999 (84/201, 41.8%), followed by <US $10,000 (47/201, 23.4%). Employment status varied, as 24.4% (49/201) of the participants were full-time employees, and 21.9% (44/201) of them were not in paid work. Regarding unmet social needs, 33.3% (67/201) of the participants found it moderately hard to meet. The majority of participants (106/201, 52.7%) were aware of and had used community resources. A substantial portion of participants primarily used the internet for discovering community resources (133/201, 66.2%), and 60.2% (121/201) of the participants were open to using a chatbot for resource finding.

**Figure 4:**
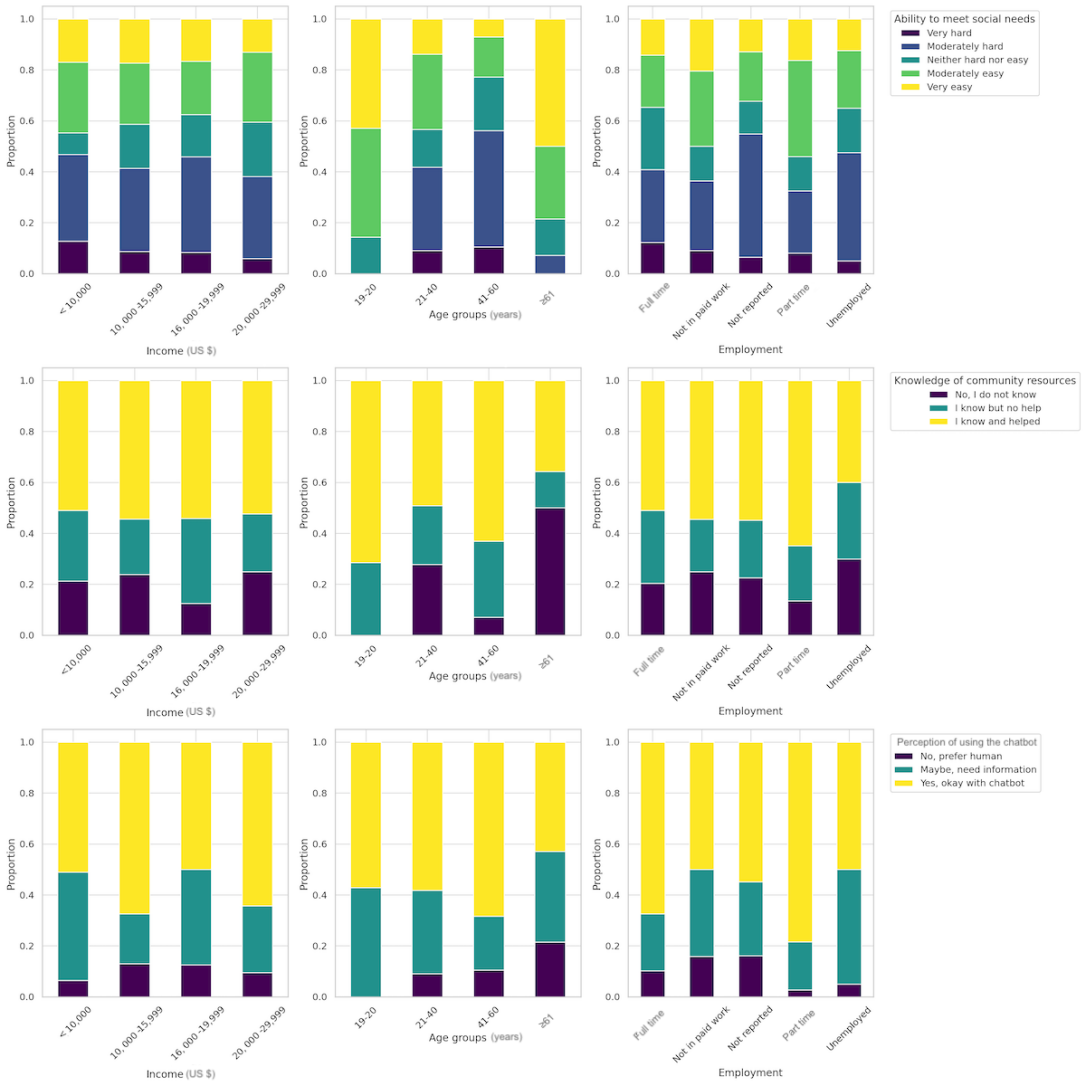
Stacked bar graphs showing the proportional distribution of the survey responses for social needs and perception toward chatbot by income, age group, and employment status.

Figure 4 outlines the proportional distribution of the responses. Lower-income groups reported a lower ability to meet social needs, with similar trends observed among middle-aged groups and those who were unemployed. There was an increase in meeting social needs among slightly higher–income earners, younger age groups, and individuals who were fully employed. Knowledge of community resources was lower among individuals who were unemployed, and the oldest age group, those aged ≥61 years, exhibited a lower level of community resource awareness and use of community programs. In terms of chatbot use, while there was a general receptiveness across all income levels, employed and younger individuals, particularly those aged between 21 and 40 years, demonstrated a higher tendency to use chatbot technology. In contrast, the oldest age group demonstrated a greater preference for human interaction over using chatbots.

**Table 1:** Demographics of web-based survey participants (n=201).

Characteristics			Participants, n (%)
**Income range (US $)**			
	<10,000	47 (23.4)	
	10,000-15,999	46 (22.9)	
	16,000-19,999	24 (11.9)	
	20,000-29,999	84 (41.8)	
**Sex**			
	Female	100 (49.8)	
	Male	100 (49.8)	
	Not reported	1 (0.5)	
**Race**			
	Asian	14 (7)	
	Black	24 (11.9)	
	White	146 (72.6)	
	Other	9 (4.5)	
	>1	8 (4)	
**Age** **group (y)**			
	19-20	7 (3.5)	
	21-40	122 (60.7)	
	41-60	57 (28.4)	
	≥61	14 (6.9)	
	Not reported	1 (0.5)	
**Employment status**			
	Full time	49 (24.4)	
	Part time	37 (18.4)	
	Unemployed (and job seeking)	40 (19.9)	
	Not in paid work (eg, homemaker, retired, or disabled)	44 (21.9)	
	Not reported	31 (15.4)	
**Ability to meet social needs**			
	Very hard	17 (8.5)	
	Moderately hard	67 (33.3)	
	Neither hard nor easy	34 (16.9)	
	Moderately easy	52 (25.9)	
	Very easy	31 (15.4)	
**Knowledge about community resources**			
	No, I do not know about these programs	45 (22.4)	
	Yes, but I could not get help	50 (24.9)	
	Yes, and they helped	106 (52.7)	
**Methods of finding community resources**			
	Internet (websites and email)	133 (66.2)	
	In person (such as at community centers and food banks)	93 (46.3)	
	Phone calls	61 (30.3)	
	Mobile apps	27 (13.4)	
	Other	20 (10)	
**Perception about using chatbot**			
	No, I would rather talk to a person	20 (10)	
	Maybe, I need to know more about how it works	60 (29.9)	
	Yes, I would be okay with using a chatbot	121 (60.2)	

The chi-square analysis did not yield strong evidence of association of income (χ^2^_6_=7.9; *P*=.24), age (χ^2^_6_=6.5; *P*=.37), and ability to meet social needs (χ^2^_8_=6.2; *P*=.63) with the perception of using a chatbot. However, there is a statistically significant association between knowledge about community resources and chatbot perception (χ^2^_4_=12.9; *P*=.01). In addition, the relationship between employment and chatbot perception is marginally close to being significant (χ^2^_8_=15.5; *P*=.051).

### Study 2: Iterative Design

**Figure 5:**
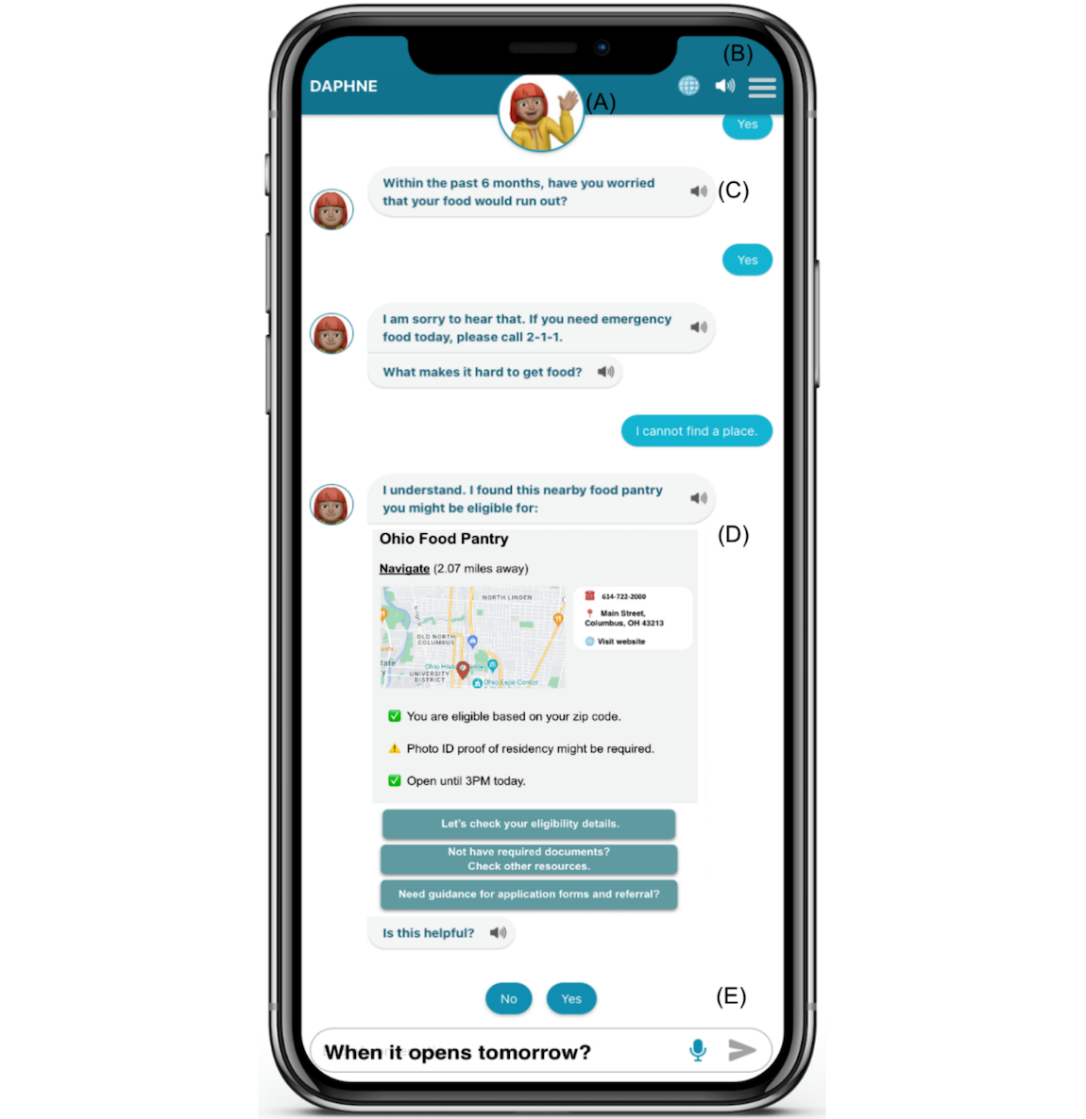
Revised semifunctional prototype (web application). (A) customizable avatar; (B) profile and setting menu, language selection, and enable or disable audio narration (text to speech); (C) repeating audio narration for preferred messages; (D) interactive resource screen with navigation and communication details and follow-up questions suggested by stakeholders; and (E) assistive buttons for quick response, text entry feature, and speech-to-text feature for voice interaction.

The themes were grouped under 3 research questions. Textbox 2 outlines the questions and themes for each question. Themes included common pain points: technology opportunities and challenges and technology considerations, including inclusivity, personalization, and information about accessing resources. On the basis of stakeholder feedback, we improved our chatbot design (Figure 5). We updated the prototype to include chatbot language options, modified language (eg, “What makes it hard to get food?”), and resource education options (ie, eligibility criteria, documentation requirements, and referral guidance). These components were initiated and are under development.

**Questions and themes from stakeholder group sessions.**

**“What are the pain points in current practices of social need screening and resource sharing?”**

- Inadequate or inconsistent screening tools: the tools used for social need screening may not be comprehensive enough for addressing all social needs associated with social determinants of health, resulting in incomplete assessments. In addition, there might be inconsistency in the use of these tools across different settings, leading to variations in the identification and understanding of social needs.
- Limited provider training and awareness: health care providers and other professionals involved in social need screening may lack sufficient training and awareness about how to implement screening, screen for unidentified needs, and address the unmet social needs. This can lead to lower quality of service as well as adversely affect the quality of life.
- Fragmented systems and lack of integration: social need screening and resource-sharing efforts are often fragmented across different divisions, departments, and organizations. This can lead to poor communication and collaboration, creating barriers to the effective identification and provision of resources.
- Insufficient resources and capacity: there may be a lack of adequate resources and capacity to address identified social needs based on the location and resources of institutions (rural vs urban health institution), resulting in unmet needs or long waiting periods for support. This can exacerbate existing disparities and negatively impact health outcomes.
- Stigma and privacy concerns: patients and families may be reluctant to disclose their sensitive information as well as their social needs due to concerns about stigma or privacy. This can prevent the accurate identification of needs and hinder access to appropriate resources.
- Cultural and linguistic barriers: cultural and linguistic differences may negatively impact communication among providers and patients or families, leading to misunderstandings and underestimation of social needs. This can result in the inadequate provision of resources and support.

**“Why should we use or not use technology to facilitate this process?”**

- Opportunities:
  Improved efficiency: technology can streamline the screening and resource-sharing process, reducing the time and effort required by both providers and patients or families. Automated systems and digital platforms can facilitate data collection, storage, and retrieval, making it easier to identify and address social needs at scale, especially within low-resource settings.
  Standardization and consistency: digital tools can help ensure that social need screening is conducted in a standardized and consistent manner across different settings, reducing variations in the identification and understanding of social needs.
  Personalization and customization: technology can enable more personalized and customized approaches to social need screening and resource sharing, tailoring interventions to the specific needs and preferences of patients and families (which can be beneficial considering cultural appropriateness and language options).
- Challenges:
  Digital divide: the use of technology may exacerbate existing disparities in access to digital tools, particularly among vulnerable populations. This can result in the further marginalization of those who may be most in need of support yet do not have access to the necessary technology.
  Privacy and security concerns: storing and sharing sensitive data and private information electronically can raise privacy and security concerns for individuals and institutions. It might be particularly concerning if technology providers have not enforced appropriate safeguards to protect the information.
  Implementation challenges: introducing a new approach using technology into social need screening and resource-sharing processes may involve significant financial and human resource investments as well as create barriers or burdens related to staff training, infrastructure, and technological compatibility.

**“How can we design and use a chatbot to connect with families in primary care settings in order to address social needs effectively?”**

- Leveraging provider and technology collaboration: this means that chatbot and health care providers (eg, primary care team) and community centers can work together to serve families better and more effectively.
- Suggested use case 1: chatbot can be used as a triage follow-up tool for health care providers and community centers to follow-up on patients after their visit to ensure that patient and family needs are met and check whether resources are useful. Thus, the chatbot can timely inform providers to intervene in case of unmet needs as well as help identify invalid or noneligible resources and update their resource list and database accordingly.
- Suggested use case 2: chatbot can be used as a prescreening tool to inform providers and community centers about what needs to be communicated with families, getting them ready for detailed conversation about the resources and how to access them. The chatbot can ease the process of support and patient engagement so that providers can timely serve more families and spare more time to engage as well as for identifying and addressing urgent needs during their conversations.
- Conversational design could be guided to be more inclusive and personal.
- Current screening instruments are not individually relatable or personal, and chatbot can be guided toward more conversational personalized screening, which can eventually inform current screening tools. Reframing dialogues toward positive attitude and social norms are some of the methods discussed.
- Cultural appropriateness and language barriers could be addressed by chatbot providing language options (eg, Ohio has a high rate of Nepali and Somali refugees with limited English proficiency and requires interpreter services) and culturally guided and appropriate conversations and dialogue flow, accounting in cultural norms and connotation (eg, In some cultures “free resource” still may mean you have to pay back due to cultural expectations or practices).
- Chatbots can help educate families about accessing resources (beyond sharing resources, guiding them on self-referral, how to check eligibility, and how to navigate web-based resources). This may eventually reduce dependency on low-risk or quickly accessible resources by families and patients, reserving time and resources of health institutions and community centers to be spent on patients and families having urgent or complex needs.

### Study 3: Prototype Evaluation

We collected feedback from 13 participants, including community health workers (n=4, 31%) and social workers (n=9, 69%), within the NCH network (Table 2). Community health workers had <5 years of professional experience (3/4, 75%). They had limited experience with chatbots and conversational agents. They are supporting families through the Connecting Families 4 Success program at NCH, which provides resource linkages to families with identified social needs.

The majority of social workers (6/9, 67%) had 1 to 10 years of experience in their current profession. Most (8/9, 89%) had prior exposure to chatbots. Social workers reported that they serve an average of 165 (SD 229) patients or families monthly. The social workers responded to the usability questions (UMUX-Lite) using a 7-point Likert scale, agreeing that the chatbot’s capabilities met expectations toward addressing social needs (average score 5.4, SD 1.1) and that it is easy to use (average score 5.6, SD 1.8). Collectively, they agreed on the usability of chatbot, providing an average score of 72 on the System Usability Scale (calculated using the regression equation developed by Lewis et al [38]).

We analyzed responses from community health workers and social workers together and grouped them under the following 4 overarching themes: user experience (how users perceived the chatbot, its functionality, and its satisfaction and interacted with it), feature preferences (user preferences for additional features in the chatbot), resource concerns (the challenges related to the accuracy, relevance, and timeliness of the resources provided by the chatbot), and perceived disadvantages and challenges (limitations and potential obstacles associated with using the chatbot; Table 3). The themes were informed by the interview and survey questions, focusing on user experience (meeting expectations and ease of use), chatbot dialogue, audio interaction, perceived advantages and disadvantages, and integration to clinical workflow. As outlined in Table 3, our thematic analysis suggests that the use of a chatbot is perceived to be useful for patients, caregivers, and providers and can help with addressing unmet social needs and resource sharing. More specifically, social workers and community health workers appreciated its clear interface but noted the need for detailed options on eligibility and documentation. Opinions on audio narration were mixed, with some valuing it for accessibility and others preferring text for privacy. The need for multilingual support and careful consideration of privacy with EHR integration was also emphasized. Users were concerned about the accuracy and currency of resource information and doubtful of the technology’s current capability to update its content without human verification. Major drawbacks included the chatbot’s inability to interpret nonverbal cues and complex situations, limited access to necessary technology for some users, and concerns about data privacy and trust.

**Table 2:** Study-3 participant demographics (n=13).

Demographics			Social worker (n=9), n (%)		Community health worker (n=4), n (%)
**Have you ever used chatbots before for any purpose?**					
	Yes, multiple times	8 (89)		1 (25)	
	No, never	1 (11)		3 (75)	
**Age group** **(y)**					
	18-24	1 (11)		2 (50)	
	25-34	3 (33)		1 (25)	
	35-44	2 (22)		1 (25)	
	45-54	3 (33)		0 (0)	
**Experience** **(y)**					
	<1	1 (11)		2 (50)	
	1-5	3 (33)		1 (25)	
	5-10	3 (33)		1 (25)	
	10-20	1 (11)		0 (0)	
	>20	1 (11)		0 (0)	

**Table 3:** Themes from interviews and web-based survey with social workers and community health workers.

Themes	Explanations	Quotes
User experience	Conversational interface was found to be simple and understandable. It was noted as “easy to use,” as it enabled turn-based conversation via natural language through SMS text messaging–like interface with the option of voice interaction. The simplicity of the dialogue was appreciated, although some suggested adding more options and details about resources, such as expanding options for eligibility, required documents, and forms. Although these components were included during the iterative design process, they were not functionally adapted in the prototype during the testing.	“[Dialogues] were clear and easy to understand” (Participant #5). “Dialogue is overall acceptable. I’d be interested in hearing what ‘other’ options it may generate for people who have food instability due to other reasons” (Participant #8).
Feature preference	There was mixed feedback on whether audio narration would be preferable. Some participants thought it would be helpful, especially for those who face language barriers or have difficulty reading or writing or physical impairment. However, others believed that families may prefer text or typing in certain cases, such as when they are in public places. Several participants mentioned the importance of offering multiple language options to engage with a diverse population, including non-English speakers. Current service limitations and inability to follow-up due to language barriers necessitate such alternative support on communicating social needs. Opinions on integrating chatbot data with EHRs^a^ were mixed. Some participants supported integration for better decision-making and follow-up, while others were concerned about privacy, consent, and the potential for surveillance or stigmatization.	“...voice to text can be sometimes challenging, as with Siri at times and this is more so common for individuals whose first language is not English” (Participant #4). “I think [chatbot] could [help if integrated to EHR] for basic medical records, but families might try to use this to ask medical questions thinking a doctor might respond” (Participant #3).
Resource concerns	Participants expressed concerns about the accuracy of resources and how to the resource information is up-to-date. The concerns were also about identifying relevant resources to specific age groups or situations, which may not be available in a single database or web resource. In the current practice, support teams (eg, community centers and social workers) need to reach out and check the availability of resources (eg, food pantry) and eligibility to ensure the resource provider is operational before referring to a patient and family. Participants emphasized their skepticism behind the need to use technology to ensure that resources are up to date (given the necessity of calling and communicating with the service providers to ensure the resource list is up to date).	“[resources] might not always be accurate if all the places are not regularly updated in the system” (Participant #9) “It will also be useful to have more details about the pantry such as open hours of operation” (Participant #6).
Perceived disadvantages and challenges	Some participants expressed concerns about the chatbot’s major communication limitation, which is its inability to assess nonverbal cues and understand nuanced situations; therefore, it may not be able to articulate social need details to guide toward appropriate resources. Limited access to technology, broadband or Wi-Fi, or mobile devices could pose challenges for some families. It is noted that some families or patients might be uncomfortable sharing personal information with a chatbot or might not trust the information provided by a chatbot.	“One disadvantage might be the chatbot not being able to assess nonverbal cues, or other concerns the family might have that can’t be typed into a box” (Participant #5). “[Chatbot] can’t share nuanced situations. Can it understand the intersection of different needs?... organizations that have food and housing resources if you are looking for orgs specifically with both” (Participant #4).

^a^EHR: electronic health record.

## Discussion

### Principal Findings

We conducted a 3-stage study with a multistakeholder group, focusing on the development and evaluation of a social need screening chatbot for families. Engaging stakeholders, including low-income households, health care providers, and family advocates, throughout the need assessment and design and development process helped identify specific needs, preferences, perceptions toward using chatbots and potential barriers to adoption. Our study highlights the importance of a multilayered and user-centered iterative design and the development of chatbots for social needs. Furthermore, it was a promising step forward to develop a chatbot collectively with partners and to serve families effectively via conversational systems [39,40]. It also contributes to the literature [19,21] by providing further evidence on diverse stakeholder perceptions of chatbot use in social need screening and resource sharing.

Our first study highlights that the awareness of community resources is traditionally less among lower-income and unemployed groups, as well as among older households. However, the majority of participants, irrespective of demographics, use the internet to identify community resources. Although this is promising, further investigation is needed on how to leverage current communication technologies to close the gap in digital inclusion, improving the awareness of resources and access [41,42]. Furthermore, a broad acceptance of chatbot technology across all income levels is observed, as younger individuals, notably those aged 21 to 40 years, lead the charge in embracing this digital interaction. This suggests a generational pivot toward using chatbots similarly to prior observations with the acceptance of new technologies [43]. The knowledge and employment status of individuals with social needs are further associated with their perception about chatbots.

Our second study highlights potential implementation areas and improvements for the chatbot to be more engaging and effective. In the current health care ecosystem, chatbots may serve a dual function as follow-up tools and triage systems, as recommended by health care providers and community centers subsequent to family visits, ensuring the effective use of resources and helping meet social needs. In line with this, chatbot data can be used for timely feedback about health care systems about unmet needs as well as to facilitate the updating of resource repositories and databases (eg, user feedback on nonoperational food pantries). When deployed as prescreening instruments, chatbots can enable providers and community centers to be adequately prepared for comprehensive discussions about resource availability and access, thereby streamlining support procedures. Conversational design can be strategically geared toward providing more inclusive and personalized need assessment [44-46]. By enhancing the relational capacities of a chatbot [47] such as showcasing positive attitudes and adhering to social norms (eg, “Others have found assistance through this local agency.”) and implementing behavioral nudges (eg, “Completing this screening will require just a few minutes.”) within the conversational design, its engagement abilities can be bolstered. In addition, a chatbot can address cultural compatibility and linguistic barriers by providing multilingual options and culturally sensitive dialogues [48]. In the context of current practices to adequately address social needs [49], chatbots can act as supportive adjuncts, supplementing and enhancing these efforts. Furthermore, chatbots can play an educational role in assisting families in understanding and accessing resources, steering them through self-referral processes, eligibility assessments, and web-based resource navigation. As a next step, chatbots can be instrumental in augmenting health literacy [50], as they have been well received in addressing social needs among populations with low literacy levels [24].

Building on stakeholder feedback, we implemented improvements in our chatbot prototype. In study 3, our descriptive analysis showed a diverse range of participants in terms of age, experience, and department affiliation, providing a rich perspective on the chatbot’s applicability with various contexts. Participants have generally rated the chatbot’s capabilities and ease of use as average to high. The results of our study indicate that the chatbot designed for addressing social needs is generally well received, with most users finding it easy to use and having a positive user experience overall. This overlaps with the current trajectory of chatbot use in health care, as the capability as well as usefulness of chatbots increases [51,52].

### Emerging Opportunities and Barriers

Audio narration emerged as a theme with mixed opinions. While some users believed it could benefit those facing language barriers or with difficulty reading, others felt that text-based communication would be more appropriate in publicly available spaces, which was also noted previously as a common concern about using voice interaction in health information exchange [53].

Resource accuracy and availability were identified as concerns by participants, emphasizing the importance of regularly updating resource information and ensuring that resources are relevant to specific age groups or profiles. To ensure the sustainability and maintenance of resources and accuracy of DAPHNE’s responses, automatic updates of the chatbot resource listings by syncing with APIs provided by established community resource platforms may ensure real-time accuracy. In addition, a user feedback mechanism is in place (regarding whether the resource is helpful) via the chatbot interface, allowing users to report any discrepancies or changes required in the resource information directly through the chatbot interface. Such a human-in-the-loop feedback mechanism is crucial for continuous improvement and helps maintain a high level of trust and reliability in the resources provided [54,55].

Integration with EHRs received mixed feedback, with some users supporting the idea for better decision-making and follow-up. Such implementation is principally viable to support decision-making with a feedback mechanism [56]. Others expressed concerns about privacy and potential stigmatization, which may lead to labeling and internalized negative stereotypes that may reduce disclosing social needs [57]. Therefore, EHR integration and adoption require detailed investigation to reduce barriers and inequality in medical documentation [57,58]. For privacy, data should be transmitted to EHRs via Health Insurance Portability and Accountability Act–compliant services and encrypted, and a governance body should be established for regular oversight [59,60]. In addition, where possible, data anonymization could be implemented to protect patient identity. To address stigma, training health care providers on handling sensitive information respectfully and confidentially is needed, which is crucial for integrating social determinants of health into patient care without bias [61].

Participants raised several disadvantages and challenges related to the chatbot’s ability to assess nonverbal cues and accurately screen needs, limited access to technology, and trust issues when sharing personal information with a nonhuman source. In this regard, using a single modality communication medium (no visual exchange) might limit the chatbot’s ability to process nonverbal cues. Multimodal approaches with chatbots may overcome this limitation in the future [62]. The chatbot’s dependency on technology platforms (computer, Wi-Fi, or smart mobile devices) and data plans may limit access. Although there are existing programs to support broadband access to low-income families (eg, Affordable Connectivity Program by Federal Communications Commission) [63] in the long term, this underscores the importance of strategic consideration of the digital divide and accessibility challenges when designing and implementing chatbots for social needs. In earlier studies, interactive voice response systems and chatbots based on SMS text messaging have been viable alternatives, which might be adapted for social need screening and resource sharing, especially in rural areas [64]. The lack of trust between the chatbot and families might negatively influence its use. Some participants expressed concerns about sharing personal information with a nonhuman actor or not trusting the information provided by the chatbot. Literature has mixed evidence toward trust between humans and chatbot [65], and further research can inform the trust built between families and chatbot.

### Expanding With Broader Social Needs

The potential scalability of the DAPHNE chatbot extends beyond its current application in food insecurity. At its current state, its design accommodates the integration of additional social needs by incorporating a flexible, rule-based conversational architecture that can be customized with minimal technical adjustments. For example, the chatbot could be adapted to screen for housing instability or transportation difficulties by updating the dialogue scripts and linking to different resource databases. Moreover, the backend infrastructure, built on cloud services, supports scalability to handle increased user traffic and data volume as the system expands.

### Improving Technical Capabilities

Although the current chatbot conversational flow was designed to be rule based, transformer-based large language models and artificial intelligence (AI)–enabled conversational agents are alternative approaches to delegate a variety of tasks [66,67]. These intelligent chatbots include a large range of functionalities that set them apart from their predecessors, such as (1) engaging in discussions across multiple topics; (2) managing multiturn conversations; (3) retaining information from previous conversations; (4) operating in both task-based and non–task-based modes; and, most importantly, (5) collaborating effectively with users. In particular, being able to work together with users, listening to their instructions, and understanding their preferences through naturally occurring conversations open up a wealth of opportunities for both health care providers and families with social needs. Although there are major concerns related to privacy, reliability, and accuracy [68], we can expect the development of hybrid solutions that have the increased conversational competence of chatbots while being constrained by the strict specifications of a rule-based system. In addition, current guidelines and practices for developing skills to engage in a sensitive conversation, such as food insecurity, can be informative for AI-enabled chatbot development [69,70]. For instance, American Hospital Association’s guidelines suggesting cultural competency, motivational interviewing, active listening, and empathic inquiry would be valuable input for conversational design and development [71].

### Future Research

It is essential to evaluate the long-term effectiveness, scale-up capability, and impact of chatbots for addressing social needs through rigorous and comprehensive evaluation methodologies [72,73]. Our study provides preliminary evidence on the iterative design and evaluation of the chatbot for addressing social needs (focusing on food insecurity screening and resource sharing with text and voice interaction). However, future research should investigate the impact of chatbot interventions on users’ health outcomes, quality of life, and access to resources, as well as the cost-effectiveness and scalability of such interventions. While chatbots can play a valuable role in addressing social needs, they are unlikely to replace human service providers entirely. Instead, chatbots can be considered complementary tools that support and enhance existing services by providing timely, personalized, and accessible information and resources. Future research should explore the potential synergies and integration opportunities between chatbots and other digital health interventions, such as telemedicine, mobile health apps, and online support groups, to maximize the overall impact on users’ health and well-being.

### Limitations

There are several limitations that should be acknowledged while interpreting the study results. First, our participants in studies 2 and 3 consist of stakeholders representing providers more than patients and caregivers, which may potentially skew the feedback toward professional perspectives. Providers may have perspectives or biased interests that differ from those of patients and caregivers, potentially leading to an overemphasis on the functionality and clinical utility of the chatbot. This skew could limit the generalizability of our findings to broader end-user experiences and might overlook key usability challenges faced by less technologically proficient users. Moreover, the diversity and size of our participant sample may not fully represent the broader population, which could limit the generalizability of our findings. Second, our research was conducted in a controlled setting with a single scenario and did not involve any real-world testing and observations. As such, the practical implications of our study remain limited to self-reported and perceived usability, feasibility, and implementation with a limited user experience. Further research in real-world scenarios is required to evaluate the effectiveness and feasibility of the chatbot in addressing social needs. Third, our research focused primarily on qualitative data, thus lacking quantitative information to assess the chatbot’s performance with a longitudinal observation. Although we collected preliminary data from households regarding their social needs and perception toward using the chatbot, the effort was limited in terms of demographic diversity and feedback (without chatbot engagement), and it may be subject to self-report bias. Future studies will aim to collect quantitative measures with real-world chatbot use, such as user logs, response accuracy, and user satisfaction rates, to provide a more comprehensive evaluation of the chatbot’s performance in addressing social needs.

### Conclusions

The study reported the iterative design and evaluation of a chatbot for social need screening and resource identification designed to scale screening and resource sharing for low-resource communities and disadvantaged neighborhoods. Furthermore, it may augment health center services, with low-risk tasks (such as resource finding and sharing) being delegated to the chatbot to scale the services provided. The DAPHNE chatbot has garnered largely favorable responses, providing initial evidence for its practicality and viability. Crucial factors in designing chatbots for social needs involve fostering user confidence, ensuring the precision of resources, and tackling accessibility obstacles. Future studies should investigate the efficacy, cost-efficiency, and expandability of chatbot initiatives, the opportunities provided by conversational AI technologies, and possible collaborations with other established digital health interventions.

## Appendix

Multimedia Appendix 1 Survey materials.

## Abbreviations

AI: artificial intelligence
API: application programming interface
DAPHNE: Dialog-Based Assistant Platform for Healthcare and Needs Ecosystem
EHR: electronic health record
NCH: Nationwide Children’s Hospital
REDCap: Research Electronic Data Capture
UMUX-Lite: Usability Metric for User Experience–Lite
