# Supplementary material for: Chatbot for Social Need Screening and Resource Sharing With Vulnerable Families: Iterative Design and Evaluation Study
Source: JMIR Hum Factors. 2024 Jul 19;11:e57114. doi: 10.2196/57114 (PMC11297373; doi:10.2196/57114)
Supplement: Multimedia Appendix 1 [file humanfactors_v11i1e57114_app1.docx]

# Survey materials - Family members

Dear Participant,

We appreciate your participation in this 4-question survey. Our aim is to understand your needs and how you access important resources like housing, transportation, financial assistance, and food. Your input will help us improve our services and technology on community programs. This survey is confidential, and your honesty is appreciated.

This survey collects demographic data and feedback on accessing essential resources. We do not gather personally identifiable information. All responses will be anonymous and stored securely for research purposes only. Participation is voluntary, and you can withdraw at any time. Your data will not be shared with third parties. Thank you for contributing to our research.

1. Think about these basic needs: food, a place to live, transportation, and financial needs. How would you rate your ability to get each of these things?
   1. Very hard
   2. Moderately hard
   3. Neither hard or easy
   4. Moderately easy
   5. Very easy
2. Do you know about local programs (like food banks, housing assistance, transportation services, financial aid, etc.) meant to help people. Can you get help from them? Please pick one:
   1. Yes, I know about these programs and they have helped me
   2. Yes, I know about these programs about I could not get help from them
   3. No, I do not know about these programs
3. How do you usually get the help or resources you need? Please pick all that apply:
   1. In person (like at community centers, food banks, etc.)
   2. Phone calls
   3. Internet (website, email)
   4. Mobile apps
   5. Other
4. Would you be okay with using a chatbot to help you find and get resources? A chatbot is like an automatic messaging or calling service that can give you information and point you to services anytime without waiting for a person to help. Please pick one:
   1. Yes, I would be okay with using a chatbot
   2. Maybe, I would need to know more about how it works
   3. No, I would rather talk to a person

# Survey materials - Social work

## Introduction:

Chatbots are intelligent computer programs that allow us to communicate with computers with written or spoken language (text or voice). They have been mostly used for customer support (e.g. banking, insurance, technical support, appointment scheduling) over mobile apps, websites or via phone calls. Here is an example chatbot from Amazon below. With the DAPHNE project at NCH, we are using a chatbot technology to communicate and understand the social needs of vulnerable or underserved families. We developed a chatbot prototype, and with

this study, our aim is to understand if we can use chatbots to connect families with

the right resources for their social needs. We kindly invite you to use our prototype

following a scenario and answer usability questions. Thank you for your

participation! We appreciate your time and feedback.

Q1: Have you ever used chatbots before for any purpose?

0 Yes, once

1 Yes, multiple times

2 No, never

Q2: Years of experience with your current profession?

0 Less than 1 year

1 1-5 years

2 5-10 years

3 10-20 years

4 More than 20 years

Q3: What is your division/department?

Q4: What is the average number of patients or families that you are caring monthly?

## Scenario

**Instructions:**

In this section, you will be introduced to a scenario for a family and asked to follow the scenario while engaging the DAPHNE chatbot app. IMPORTANT NOTE DAPHNE app is a limited prototype that is aiming to demonstrate food insecurity screening as part of social needs and provide responses with resources at this stage. It is not yet complete to assist with other social needs and the images/links may not be functional. It may fail to respond to any other questions at the moment. Some functions will be available in future versions including voice entry, language selection and conversation capability.

**Scenario:**

Please assume you are Sam, parent of a child who had a primary care visit earlier this year. During the visit, you have indicated that you have had trouble accessing food or finding eligible food pantries nearby last year. Then, you have been introduced to the DAPHNE chatbot app to provide external support out of the clinic. Now, you have access to the DAPHNE app over your phone, tablet PC or computer. DAPHNE is assigned to communicate with you and share resources. Please use this link to access the app: Link for chatbot app or please use <web link> [App will open in a new browser window. There will be audio narration (please unmute your computer). You may refresh the app page to restart the app]

Please locate the language selection menu (language options are not active currently). The chatbot is able to narrate conversations. You can use the audio toggle button to turn on or off the narration function. Microphone button on the text box is to help respond via speech (not active currently). If you would like to restart screening conversation, you can use the refresh button to restart. Scenario tasks:
1. Please enter the link to your web browser or click the link for the chatbot app.

2. Select or enter "OK" to initiate screening and confirm your zip code entering "Yes"

3. Select or enter "Yes" for food insecurity screening question

4. To elaborate, select "I cannot find a place"

5. Review the response screen and information provided (the screen is not interactive but an image currently. It will be an interactive screen in the following versions of the chatbot. Please assume the map and other links are functional).

6. Select or enter "Yes" for the next question about resources being helpful, and end the session.

7. Please restart the session (refresh page) and follow the steps 1 to 4. At step 4, select reason: "I cannot afford it" or "other" to enter another reason.

8. Review the response. Select or enter "No" for the next question about resources being helpful and review the response.

9. Review the response and end the session

## Usability feedback

UMUX-1: The chatbot capabilities meet the requirements to address social needs (accessing information for resources):

[0 Strongly Disagree 1 Disagree 2 Somewhat disagree 3 Neither agree nor disagree 4 Somewhat agree 5 Agree 6 Strongly Agree]

UMUX-2: The chatbot is easy to use:

[0 Strongly Disagree 1 Disagree 2 Somewhat disagree 3 Neither agree nor disagree 4 Somewhat agree 5 Agree 6 Strongly Agree]

## Open-ended questions

Do you think the flow of chatbot dialogues, questions and responses are acceptable? Please share your thoughts and suggestions on how to improve the dialogue towards helping families with social needs.

Do you think having audio narration and responding by talking (not functional in this prototype) may be preferable by the families? Please share your thoughts.

Please share advantages or opportunities you think about using the chatbot for social needs screening and sharing resources?

Please share disadvantages or shortcomings you think about using the chatbot for social needs screening and sharing resources?

Do you think the chatbot data (identified social needs and provided resources per patient or family) should be integrated to patient medical records in the EHR system? Please share your opinion and vision.

Please provide any additional comments or suggestions about this chatbot to be a more effective and impactful tool for families.
